# Tranexamic acid for safer surgery: the time is now

**DOI:** 10.1093/bjs/znac252

**Published:** 2022-09-05

**Authors:** The UK Royal Colleges Tranexamic Acid in Surgery Implementation Group, Michael P W Grocott, Mike Murphy, Ian Roberts, Rob Sayers, Cheng-Hock Toh

**Affiliations:** Royal College of Anaesthetists, London, UK; NIHR Southampton Biomedical Research Centre, University Hospital Southampton/University of Southampton, Southampton, UK; NHS Blood and Transplant, Oxford University Hospitals NHS Foundation Trust and the University of Oxford, Oxford, UK; Clinical Trials Unit, London School of Hygiene and Tropical Medicine, London, UK; University of Leicester, Leicester, UK; Royal College of Surgeons of England, London, UK; University of Liverpool, Liverpool, UK; Royal College of Physicians, London, UK

There are about 310 million major surgical procedures performed each year worldwide, with more than 4 million deaths within 30 days postoperatively^1^. Nearly 8 million surgical procedures are carried out each year in the UK^1^. Although surgical mortality appears to be decreasing in Britain, ∼85 000 people die within 30 days, increasing to 178 000 deaths within 90 days^2^. Major bleeding is strongly associated with 30-day mortality, accounting for the largest share (16 per cent) of deaths^3^. Major bleeding, mostly on the day of surgery, is more common than sepsis. For this reason, the strong evidence that tranexamic acid substantially reduces the risk of surgical bleeding is important to anaesthetists, surgeons, patients, and healthcare systems.

Tranexamic acid reduces bleeding by inhibiting the proteolysis of fibrin blood clots by plasmin. Evidence that tranexamic acid prevents surgical bleeding, reducing the need for blood transfusion and for reoperation because of bleeding, has been available for a decade^4,5^, but uncertainty about the risk of thromboembolic events have limited its use. Large randomized trials in trauma (20 211 patients) and postpartum haemorrhage (20 060 patients) show that tranexamic acid significantly reduces bleeding deaths without increasing the risk of thrombosis^6,7^. Recent meta-analyses of randomized trials show no increased risk of thromboembolic events with tranexamic acid (risk ratio (RR)=1.00; 95 per cent c.i. 0.93–1.08). Although these analyses included clinical trial data on more than 100 000 patients, many of the trials were small, which limits the reliability of the conclusions^8,9^. However, the larger (RR=0.96; 95 per cent c.i. 0.85–1.07) and high-quality trials (RR=0.98; 95 per cent c.i. 0.90–1.06) also showed no evidence of increased risk.

The recent publication of the POISE-3 (Peri-Operative Ischemic Evaluation-3) trial is a major contribution to our understanding of the benefits and risks of tranexamic acid in surgery, and deserves urgent attention^10^. The POISE-3 trial randomly allocated 9535 adults at risk of bleeding and cardiovascular complications undergoing noncardiac surgery to receive tranexamic acid or matching placebo. It found that tranexamic acid reduces major bleeding by ∼25 per cent and significantly reduces blood transfusion. The reduction in major bleeding was similar regardless of type of surgery, also in keeping with previous results.

The primary safety outcome in the POISE-3 trial was a composite of myocardial injury, non-haemorrhagic stroke, peripheral arterial thrombosis, and symptomatic proximal venous thromboembolism. This outcome occurred in 14.2 per cent of patients in the tranexamic acid group and 13.9 per cent patients in the placebo group (hazard risk [HR]=1.02; 95 per cent c.i. 0.92–1.14) indicating a low probability of a small (0.3 per cent) increase in risk. Before the POISE-3 trial, the ATACAS (Aspirin and Tranexamic Acid for Coronary Artery Surgery) trial was the largest trial of tranexamic acid in surgery^11^. A total of 4661 patients scheduled to have coronary artery bypass surgery and at risk for perioperative complications were randomly allocated to receive tranexamic acid or placebo. The primary outcome, a composite of death and thrombotic complications within 30 days of surgery, occurred in 16.7 per cent of the tranexamic acid group and in 18.1 per cent of the placebo group (RR=0.92; 95 per cent c.i. 0.81–1.05), indicating a low probability of a small decrease (1.4 per cent) in risk. The ATACAS trial also found that tranexamic acid reduced the risk of reoperation because of major bleeding and reduced receipt of a blood transfusion.

For reasons of statistical power, it is almost impossible to detect a small increase or decrease in the risk of thrombosis with tranexamic acid, even in clinical trials in high-risk patients. Because bleeding is common and thromboembolic events are comparatively rare, in our opinion the balance of benefits and risks clearly favours the use of tranexamic acid. It may seem counterintuitive to give both tranexamic acid to reduce surgical bleeding and anticoagulants to prevent venous thrombosis. However, we need to protect patients from both of these complications, and based on the available clinical trial evidence, tranexamic acid does not appear to increase the risk of thrombosis^8,9^.

Tranexamic acid is inexpensive and its use in surgery is cost-effective even when resources are severely limited^12^. The reduction in surgical blood transfusion has important implications for transfusion-transmitted infections, especially in sub-Saharan Africa. In addition to improving patient outcomes, wider use of tranexamic acid in surgery would reduce transfusion-related risks, notably human immunodeficiency virus (HIV), hepatitis B and C, and risks from newly emerging viruses^13^. Surveys show that patients and healthcare providers would prefer to avoid bleeding and the need for blood transfusion^14^.

Tranexamic acid for ‘adults who are having surgery and are expected to have moderate (>500 ml) blood loss’ has been a National Institute for Health and Care Excellence (NICE) quality standard since 2016^15^. However, a recent UK National Health Service Blood & Transplant (NHSBT) national comparative audit of compliance with this standard showed that at least one-third of patients undergoing such surgery did not receive it^16^. We estimate that compliance with the quality standard would prevent more than 15 000 major surgical bleeds, and save 33 000 units of blood and many millions of pounds for the NHS each year. To reap these benefits, we have established an implementation group with representation from the Royal College of Surgeons of England, the Royal College of Anaesthetists, and the Royal College of Physicians. It is not our role to dictate how individual surgical patients should be treated. Our aim is to make sure that all surgeons and anaesthetists are aware of the benefits of tranexamic acid use in surgery. We suggest that tranexamic acid use is considered in all adults having in-patient surgery and that ‘consideration of tranexamic acid use’ is included in the Surgical Safety Checklist of all hospitals.

We plan to disseminate evidence about tranexamic acid in surgery in journals, websites, via the UK Federation of Surgical Specialty Associations, and on social media with short video clips. We have sought the support of our trainee (surgical, anaesthetic, and haematology) networks to spearhead local leadership and will work with implementation scientists and the NHSBT audit programme on audit, feedback, and benchmarking. The NHS has a system in which effective care is incentivized by per-patient payments to hospitals. This system helped to increase tranexamic acid use in trauma after the CRASH-2 (Clinical Randomisation of an Antifibrinolytic in Significant Haemorrhage-2) trial and we will try to use this approach as well^17^. We hope that the relevant professional groups in other countries will take similar steps since this should be a global effort. To this end, we have asked the WHO to consider inclusion of tranexamic acid on the WHO Surgical Safety Checklist. Wider use of tranexamic acid will improve surgical safety, reduce unnecessary blood use, and release funds for other purposes within the healthcare system. We have the evidence, we now need to act on it.

##  

*Disclosure*. The authors declare no conflict of interest.

## References

[1] Weiser T.G. , Haynes A.B., Molina G., Lipsitz S.R., Esquivel M.M., Uribe-Leitz T., et al Estimate of the global volume of surgery in 2012: an assessment supporting improved health outcomes. Lancet 2015; 385: S11 10.1016/S0140-6736(15)60806-6 26313057

[2] Abbott T.E.F. , Fowler A.J., Dobbs T.D., Harrison E.M., Gillies M.A., Pearse R.M. Frequency of surgical treatment and related hospital procedures in the UK: a national ecological study using hospital episode statistics. Br J Anaesth 2017; 119: 249–257 28854546 10.1093/bja/aex137

[3] Vascular Events in Noncardiac Surgery Patients Cohort Evaluation (VISION) Study Investigators, Spence J., LeManach Y., Chan M.T.V., et al Association between complications and death within 30 days after noncardiac surgery. CMAJ 2019; 191: E830–E837 31358597 10.1503/cmaj.190221 PMC6663503

[4] Ker K. , Edwards P., Perel P., Shakur H., Roberts I. Effect of tranexamic acid on surgical bleeding: systematic review and cumulative meta-analysis. BMJ 2012; 344: e3054 22611164 10.1136/bmj.e3054 PMC3356857

[5] Ker K. , Prieto-Merino D., Roberts I. Systematic review, meta-analysis and meta-regression of the effect of tranexamic acid on surgical blood loss. Br J Surg 2013; 100: 1271–1279 23839785 10.1002/bjs.9193

[6] The CRASH-2 trial collaborators, Shaku H., Roberts I., Bautista R., Caballero J., Coats T., Dewan Y., et al Effects of tranexamic acid on death, vascular occlusive events, and blood transfusion in trauma patients with significant haemorrhage (CRASH-2): a randomised, placebo-controlled trial. Lancet 2010; 376: 23–32 20554319 10.1016/S0140-6736(10)60835-5

[7] WOMAN Trial Collaborators . Effect of early administration of tranexamic acid on mortality, hysterectomy, other morbidities in women with postpartum haemorrhage (The WOMAN trial): a randomised, placebo-controlled trial. Lancet 2017; 389: 2105–2116 28456509 10.1016/S0140-6736(17)30638-4 PMC5446563

[8] Taeuber I. , Weibel S., Herrmann E., Neef V., Schlesinger T., Kranke P., et al Association of intravenous tranexamic acid with thromboembolic events and mortality: a systematic review, meta-analysis, and meta-regression. JAMA Surg 2021; 156: e210884 33851983 10.1001/jamasurg.2021.0884 PMC8047805

[9] Murao S. , Nakata H., Roberts I., Yamakawa K. Effect of tranexamic acid on thrombotic events and seizures in bleeding patients: a systematic review and meta-analysis. Crit Care 2021; 25: 380 34724964 10.1186/s13054-021-03799-9 PMC8561958

[10] Devereaux P.J. , Marcucci M., Painter T.W., Conen D., Lomivorotov V., Sessler D.I., et al Tranexamic acid in patients undergoing noncardiac surgery. N Engl J Med 2022; 386: 1986–1997 35363452 10.1056/NEJMoa2201171

[11] Myles P.S. , Smith J.A., Forbes A. Silbert B., Jayarajah M., Painter T., et al Tranexamic acid in patients undergoing coronary-artery surgery. N Engl J Med 2017; 376: 136–148 27774838 10.1056/NEJMoa1606424

[12] Guerriero C. , Cairns J., Jayaraman S., Roberts I., Perel P., Shakur H. Giving tranexamic acid to reduce surgical bleeding in sub-Saharan Africa: an economic evaluation. Cost Eff Resour Alloc 2010; 8: 1 20163726 10.1186/1478-7547-8-1 PMC2832621

[13] Jayaraman S. , Chalabi Z., Perel P., Guerriero C., Roberts I. The risk of transfusion-transmitted infections in sub-Saharan Africa. Transfusion 2010; 50: 433–442 19843290 10.1111/j.1537-2995.2009.002402.x

[14] Vetter T.R. , Adhami L.F., Porterfield J.R. Jr., Marques M.B. Perceptions about blood transfusion: a survey of surgical patients and their anesthesiologists and surgeons. Anesth Analg 2014; 118: 1301–1308 24842177 10.1213/ANE.0000000000000131

[15] NICE quality standards for blood transfusion 2016. Available from: https://www.nice.org.uk/guidance/qs138. (Accessed 13 July 2022).

[16] NHS Blood & Transplant . National comparative audit of NICE quality standards for transfusion. Available from: https://hospital.blood.co.uk/audits/national-comparative-audit/national-comparative-audit-reports/. (Accessed 13 July 2022).

[17] Coats T.J. , Fragoso-Iñiguez M., Roberts I. Implementation of tranexamic acid for bleeding trauma patients: a longitudinal and cross-sectional study. Emerg Med J 2019; 36: 78–81 30530744 10.1136/emermed-2018-207693

